# Age-driven modulation of tRNA-derived fragments in *Drosophila* and their potential targets

**DOI:** 10.1186/s13062-015-0081-6

**Published:** 2015-09-16

**Authors:** Spyros Karaiskos, Ammar S. Naqvi, Karl E. Swanson, Andrey Grigoriev

**Affiliations:** Department of BiologyCenter for Computational and Integrative Biology, Rutgers University, Camden, NJ 08102 USA

**Keywords:** RISC, Argonaute, Aging, Small RNA, ncRNA, tRNA, tRF

## Abstract

**Background:**

Development of sequencing technologies and supporting computation enable discovery of small RNA molecules that previously escaped detection or were ignored due to low count numbers. While the focus in the analysis of small RNA libraries has been primarily on microRNAs (miRNAs), recent studies have reported findings of fragments of transfer RNAs (tRFs) across a range of organisms.

**Results:**

Here we describe *Drosophila melanogaster* tRFs, which appear to have a number of structural and functional features similar to those of miRNAs but are less abundant. As is the case with miRNAs, (i) tRFs seem to have distinct isoforms preferentially originating from 5’ or 3’ end of a precursor molecule (in this case, tRNA), (ii) ends of tRFs appear to contain short “seed” sequences matching conserved regions across 12 *Drosophila* genomes, preferentially in 3’ UTRs but also in introns and exons; (iii) tRFs display specific isoform loading into Ago1 and Ago2 and thus likely function in RISC complexes; (iii) levels of loading in Ago1 and Ago2 differ considerably; and (iv) both tRF expression and loading appear to be age-dependent, indicating potential regulatory changes from young to adult organisms.

**Conclusions:**

We found that *Drosophila* tRF reads mapped to both nuclear and mitochondrial tRNA genes for all 20 amino acids, while previous studies have usually reported fragments from only a few tRNAs. These tRFs show a number of similarities with miRNAs, including seed sequences. Based on complementarity with conserved *Drosophila* regions we identified such seed sequences and their possible targets with matches in the 3’UTR regions. Strikingly, the potential target genes of the most abundant tRFs show significant Gene Ontology enrichment in development and neuronal function. The latter suggests that involvement of tRFs in the RNA interfering pathway may play a role in brain activity or brain changes with age.

**Reviewers:**

This article was reviewed by Eugene Koonin, Neil Smalheiser and Alexander Kel.

**Electronic supplementary material:**

The online version of this article (doi:10.1186/s13062-015-0081-6) contains supplementary material, which is available to authorized users.

## Background

Transfer RNAs (tRNAs) have been traditionally seen as key players in protein translation, but recently there have been multiple attempts to understand them as regulatory molecules [1–3]. There are two main species of tRNA-derived small RNAs that are categorized based on length and biogenesis, including tRNA-derived small RNAs (tsRNAs, ~28-40 nt) and tRNA-derived fragments (tRFs, ~16-24 nt) [4, 5]. In this study, we focus specifically on tRFs, represented by three different fragment types based on cleavage pattern. One type is produced from the tRNA 5’ part (ending before the anticodon loop), while the other two types originate from the 3’ region, and contain either multiple uracils or a CCA modification at the end [2, 6, 7]. There have been various attempts to determine the biogenesis pathways and potential cleavage events that make these tRFs distinct from one another [1, 6–11].

Previous studies have demonstrated regulatory function of these tRFs by postulating that they bind and repress mRNAs in a fashion similar to microRNAs (miRNAs) or even compete with miRNAs [2, 5, 7, 9, 12–14]. It is unclear if they act like plant miRNAs that are fully complementary to their targets, or like animal miRNAs that have a specific pairing “seed” region. Conflicting models of such seed regions have been proposed. One of them has suggested a traditional miRNA-like silencing based on complementarity of the 5' seed sequence of a tRF to a short sub-sequence within a 3’ UTR of a transcript [10]; another has shown that the last 8–10 nucleotides (nts) on the 3’ end of the tRF in the 5’ portion of the full tRNA are responsible for mRNA repression [15].

In the present study, we elucidated tRF/mRNA pairing further by developing a computational approach and a pipeline analogous to miRNA seed-pairing studies [16–18]. Searching for conserved regions among 12 *Drosophila* species, we predicted tRF seeds and hybridization patterns similar to that of miRNAs. In a striking parallel to the experimental observations, we also found cases of both 3’- and 5’-located potential seeds for different tRF species. Some of the functions of tsRNAs/tRFs have been connected to stress, metabolism, and differentiation suggesting the species may be critical regulatory molecules for proper cellular growth and maintenance [3, 7–10, 12, 15, 19, 20]. Expanding this functional catalog in our study, we observed significant enrichment in neuronal function and development among potential targets of the prominent tRF isoforms.

We further analyzed the association with age. Recent studies have highlighted that miRNAs are associated with the aging process, showing differential isoform expression and differential RISC loading of specific miRNAs with age, related to modifications on the 3’ end, including untemplated additions, 2’-O-methylation or imprecise Drosha/Dicer cleavages [21, 22]. Here, we present a follow-up computational analysis of the same deep-sequencing libraries, this time focusing on tRFs originating from multiple tRNAs. In addition to the *in silico* prediction of seed regions, we examined changes in individual tRF isoforms with age. This unexpectedly revealed diverse patterns, resembling those of miRNA and suggesting that tRFs may impact age-associated events, while simultaneously being modulated with age. Taken together, these findings suggest that despite the lower counts in deep-sequencing experiments, tRFs represent not degradation products but potentially important players in Argonaute pathways, expanding their role as regulatory molecules.

## Results

Using four different *D. melanogaster* small RNA libraries, including co-immunopreciptations of Ago1 and Ago2 in flies aged 3 days and 30 days [21], we observed striking patterns of age-dependent expression, structure and preferential loading of tRFs into RISC complexes. Following the similarity of tRF features with miRNAs, we predicted potential targets for further experimental validation that would be the ultimate test of the biological functionality of tRFs.

### Read distributions of tRNA fragments are similar to miRNAs

The read distributions mapping to known miRNAs usually show an asymmetry favoring the mature arm of a given miRNA stem-loop sequence, usually seen as a high relative frequency of the reads aligning to one of the arms (5’ or 3’). At times, reads that originate from the middle or miRNA loop section are observed, typically with a very low frequency.

We investigated whether tRF-tRNA alignments displayed similar patterns to miRNAs in the read distributions. First, we found that tRF reads, which were more abundant in the Ago2 libraries, mapped to >100 nuclear and mitochondrial *Drosophila* tRNA genes covering the whole spectrum of 20 amino acids. This is in contrast to previous studies, which have usually reported fragments from only a few tRNAs [10, 15, 20, 23]. We also observed multiple isoforms of the same tRF being expressed. Interestingly, these mappings showed very specific patterns: the reads typically aligned to either the 5’ or 3’ region of the tRNA molecule, and often had identical start positions or presumed cleavage sites (see below). One of the tRF ends in these cases matched the respective end of the host tRNA, while the other showed some variability comparable to that observed in miRNA [21, 22]. In other words, the distribution of reads that mapped appeared as non-random and precise as those of miRNAs, strongly suggesting that their source was not indiscriminate degradation but rather a targeted biological process.

**Fig. 1:**
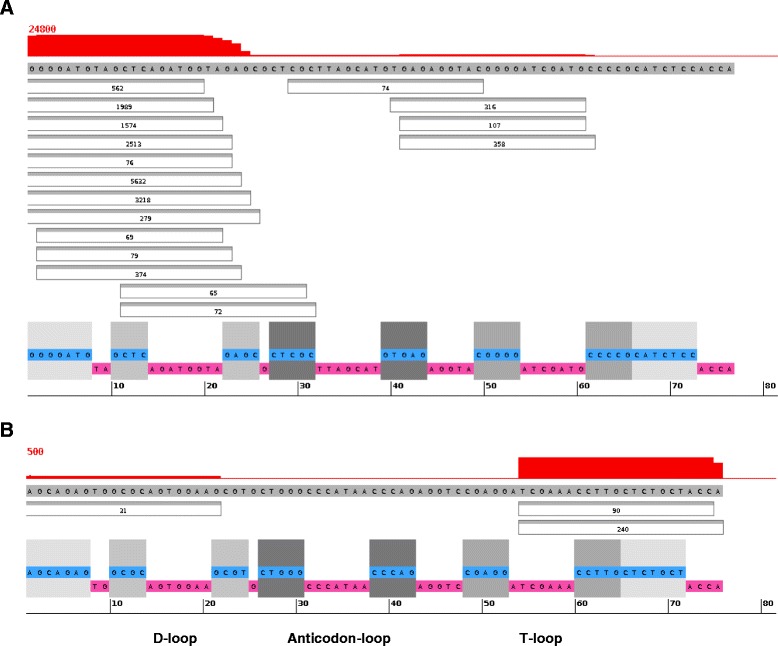
Examples of Read Distribution Patterns of tRFs. Screenshot of our RNA display, showing reads that align to tRNA-Ala in the Ago2, 30 days library (**a**) and MetCAT in Ago1, 30 days (**b**). Sequence at the bottom with the magenta background indicates single-stranded (loop) regions in the tRNA molecule, while the cyan background and matching grey boxes indicate stems. The red on top indicates read depth coverage of specific regions of the tRNA. Reads (boxes in the middle) with counts of at least 1 % of the most abundant read are displayed; lower count reads are omitted for compact visualization

All detected *Drosophila* tRFs and their relative read distributions in visual format can be found on our website [24]; here we illustrate the findings with the two examples of tRFs of different level of abundance, AlaAGC and MetCAT tRFs (Fig. 1). As was typical for most tRFs, the read distributions invite comparisons to a canonical miRNA structure, suggesting that specific cleavage mechanisms may be at work. We observed clearly defined boundaries for 5’ and 3’ regions. The uneven read distribution allows one to speculate that, in case of AlaAGC (Fig. 1a), the 5’ arm is the analog of a miRNA mature and/or functional strand, while the 3’ arm is similar to a passenger strand (that would eventually be degraded). The low frequency of reads mapping to the middle region is akin to miRNA loop regions. MetCAT is an example of the opposite case of prevalent read counts in the 3’-end (Fig. 1b). Generally, the majority of tRF reads showed a miRNA-like asymmetric distribution by aligning to either the 5’ or 3’ region of the tRNAs. This type of visualization is particularly useful because it may shed light into potential 5’ or 3’ modifications, which may include alternative cleavage sites, deletions, non-templated additions, and RNA editing events [25–27].

### Age-associated Global Shift of Ago1 vs Ago2-loaded tRFs

A number of further similarities to miRNAs were suggested by the association of the tRFs with the Argonaute proteins (Ago1 and Ago2) of the two RISC complexes. Previously, we have analyzed Ago1 and Ago2 loading of microRNAs and found age-specific patterns [21].

As with miRNAs, we observed that the total levels of Ago-loaded tRFs changed with age. In Ago1 the normalized read counts for 3 days and 30 days stayed relatively constant at ~5,000. In contrast, in Ago2 there was a 4-fold increase (from 5,000 to ~20,0000 normalized total read counts) between 3 to 30 days. Amongst tRFs with counts >100 (arbitrary threshold for illustrative purposes), 8 were downregulated and 4 upregulated in Ago1, while all 40 Ago2-associated tRFs were upregulated with age, indicating possible functional importance in an age-related manner.

**Fig. 2:**
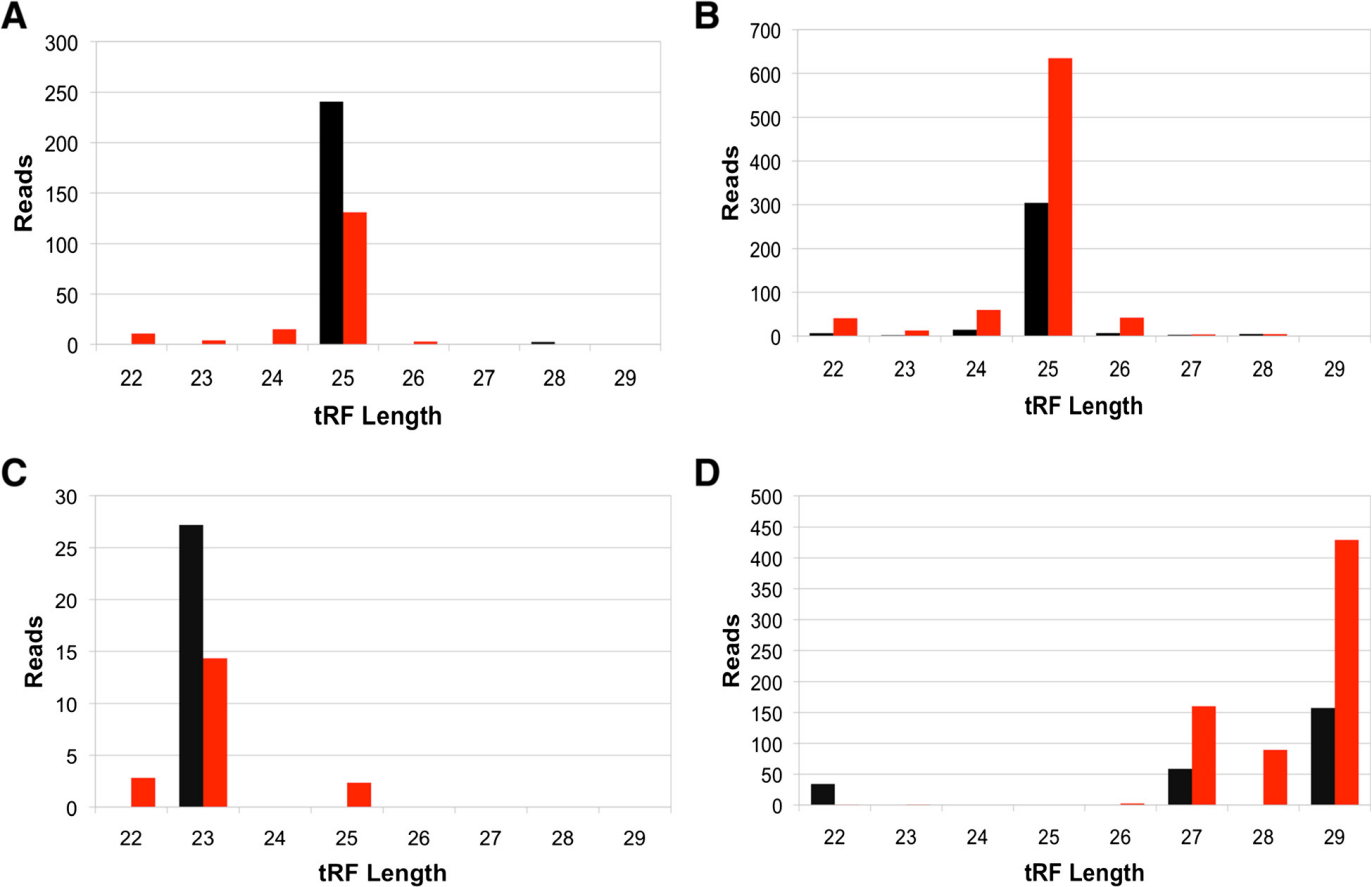
Distinct tRF Isoform Changes with Age. Isoform distributions for GluCTC in **a** Ago1-IP and **b** Ago2-IP, **c** AspGTC in Ago1-IP and **d** Ago2-IP libraries. Both tRFs show a decrease in Ago1, but an increase of specific isoforms in Ago2 with age. Black bars represent normalized counts at 3 days, while red bars represent normalized counts at 30 days. The same GluCTC is also presented in Fig. 3 (GluCTC-2)

Further investigating this result, we determined whether the differences in loading into Ago2 reflected an increased association of specific isoforms over others. This particular phenomenon is seen in miRNAs [21], so it was of interest to assess if there was a similarity in tRF behavior. We first identified two tRFs, GluCTC and AspGTC, that displayed multiple isoforms in both the Ago1-IP and Ago2-IP libraries and that also showed differential loading with age, with the most abundant isoform changing two-fold or more (Fig. 2). For GluCTC we observed the same isoform, the 25mer, being loaded onto both RISC complexes, but in Ago1 it showed a decrease with age, while in Ago2 it showed an increase with age, hinting at a mechanism that either actively partitions these fragments at the loading step in the biogenesis pathway or contributes to their retention with age when loaded to Ago2 (Fig. 2a-b). In the case of AspGTC, the isoform (29mer) that is most abundant was not detected at all in Ago1, while it was readily loaded into Ago2, which also showed increased loading with age (Fig. 2c-d).

**Fig. 3:**
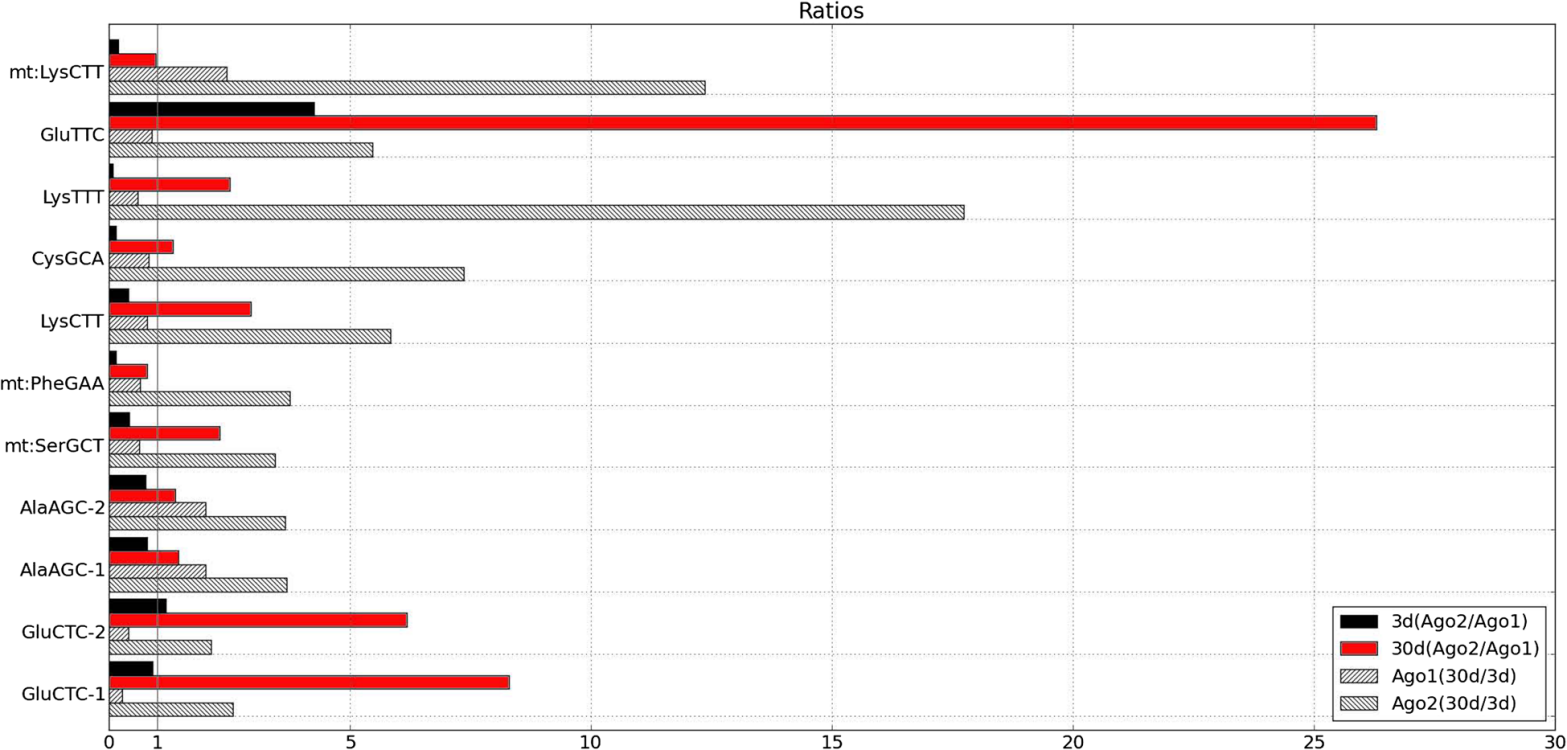
Differential and Preferential Loading. Plot of abundant tRFs that are present in all four libraries. Plots show relative ratios of reads: Ago2 to Ago1 in 3 days (black) and 30 days (red); 30 days to 3 days in Ago1 (slash) and Ago2 (back slash)

Further, we considered loading ratios of 30 days to 3 days for each tRF. Our findings indicated that loading onto Ago2 increased at 30 days, while Ago1 loading decreased or stayed the same as at 3 days (Fig. 3). Not all the tRFs are shown: e.g., Gly-related ones did not have any reads in the Ago1 libraries, and no reads were found in Ago1 for the major Ago2 isoform of AspGTC tRF depicted in Fig. 2. In several cases, distinct fragments from different tRNA genes with the same anticodon were detected, e.g., for GluCTC. When tRF sequences allowed us to distinguish such tRNA genes, we named them tRNAgene-1, tRNAgene-2, and so on (note that a union of all GluCTC isoforms in Fig. 2 corresponds to GluCTC-2 in Fig. 3). When tRF sequences could be assigned to more than one of such tRNA genes, we assumed all of these genes contributed equally to the observed tRF counts.

We then examined tRFs that were both Ago1- and Ago2-loaded in order to ascertain any age preference. We specifically looked at tRFs at the two different time points and compared their ratios in Ago2- and Ago1-associated libraries (Fig. 3). At 3 days, we observe that the ratios are either below 1 or very close to 1, with the exception of GluTTC. Thus at 3 days, there is either a preference for Ago1 or no preference at all. However, at 30 days the reverse is the case: for most tRFs we detected at least a two-fold increase in Ago2 loading. Hence, tRFs are more likely to be loaded onto Ago2 and not Ago1 in older flies, confirming an age preference amongst loaded tRFs.

We next focused specifically on the tRF species containing CCA at the 3’-end and examined their accumulation with age in both Ago1 and Ago2. Such species showed a two-fold increase in Ago1 libraries (6 % to 12 %) from 3 days to 30 days, and even higher in Ago2 libraries (6 % to 16 %), supporting the notion that fragments of mature tRNAs contribute to the global increase of loading with age.

Together, these data support the idea that the loading patterns of tRFs between Ago1 and Ago2 change dramatically with age, such that Ago2-loading of select isoforms increases, while Ago1-loading of tRF isoforms belonging to the same tRNA decreases. These results are similar to findings of age-dependent loading of miRNAs [21] and they also indicate that there may be distinct pathways for Ago loading by recognizing, partitioning or retaining specific isoforms, which may change as a function of age.

### Seed sequences in conserved regions

The mechanism of tRF action upon loading into Ago1 and Ago2 still remains unclear, but there are clues to suggest a miRNA-like pathway of execution. For example, the fragments have been detected in the cytoplasmic fraction of cells [15], several studies have shown trans-silencing capabilities of tRFs, and the silencing of a mock mRNA fully complementary to a tRF [14, 28] has been demonstrated. Some authors [10, 20] suggest a traditional miRNA-like silencing based on complementarity of the 5’ seed sequence of a tRF to a short sub-sequence within a 3’ UTR of a transcript [16–18]. Another study, however, suggests a 3’ seed sequence, while ruling out a 5’ or a mid-tRF seed binding [15].

**Fig. 4:**
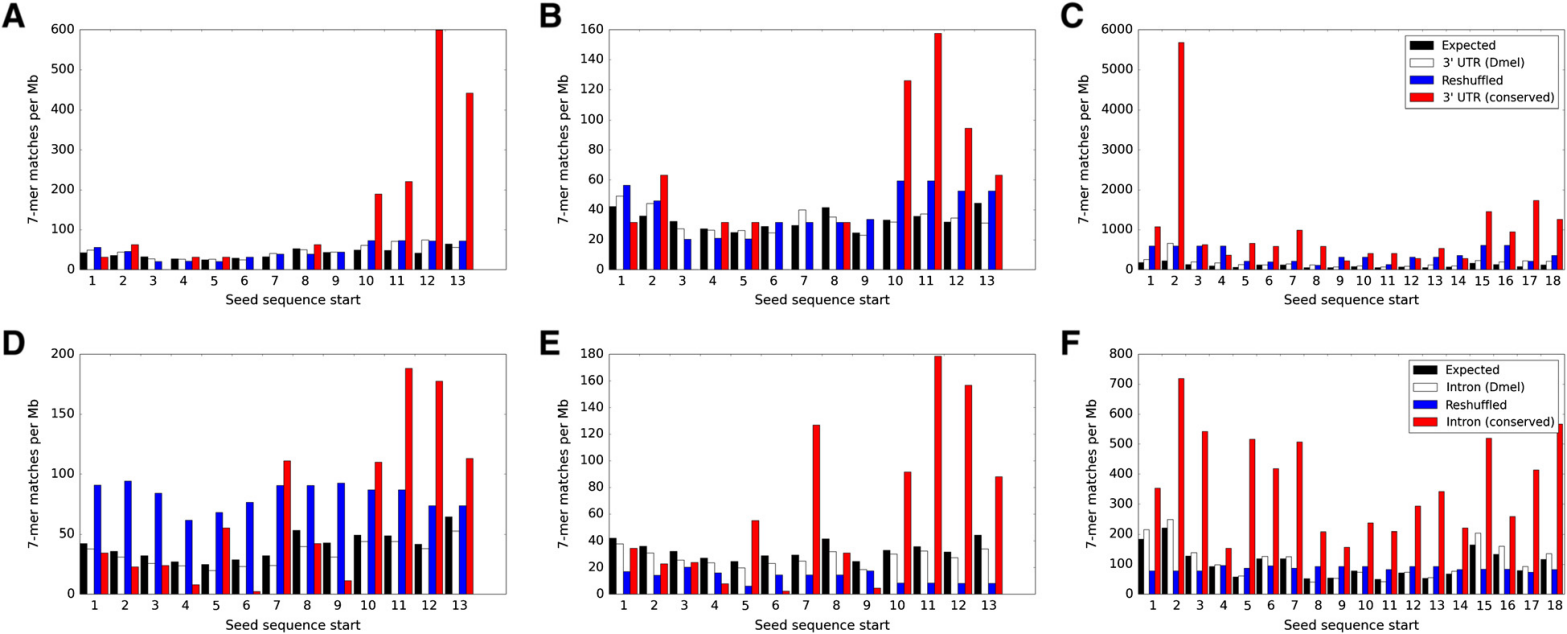
Candidate Seed Regions for tRFs. The numbers of sequence matches in the 3’ UTR regions (**a**-**c**) and introns (**d**-**f**) are plotted vs window start positions of 7mer windows of (**a**, **d**) GlyGCC, (**b**, **e**) GlyTCC and (**c**, **f**) mt:SerGCT tRFs in *Drosophila*. Color key for the top row is given in (**c**), for the bottom row in (**f**). Expected number of matches is shown in *black* and average number of matches for all other 7mers with the same nucleotide composition as the given window is shown in *blue*. The observed number of matches in the *D. melanogaster* genome is shown in *white* and in the conserved regions of 12 *Drosophila* genomes is shown in *red*

To further explore the notion of mRNA-targeting, we developed a computational pipeline to detect a potential location of a seed sequence (analogous to that of animal miRNAs) in the tRFs. In miRNAs, 3’-compensatory sites [29] and central pairing sites [30] have been reported in addition to the most prevalent 5’ seeds [15–18]. For seed finding we followed the same approach used to identify the seed sequence in microRNAs [15–18], with short sequence windows sliding along the tRF sequence, without any location constraints. Then we found exact matches of the reverse complements of these sequence windows to the 5’ UTR, 3’ UTR, intron and exon (CDS) sequences in the *D. melanogaster* genome and in the conserved portions of these regions of the 12 *Drosophila* genomes [31]. For a window of length *k* we then compared the observed match counts with those expected by chance (estimated from *k*-mer genomic frequency) and with the mean frequency of all other possible *k*-mer sequences produced by reshuffling the nucleotides in the window. In agreement with our conjecture that tRFs may harbor miRNA-like short seed sequences, 7-nt windows showed good discrimination between the conservation levels of 5’ and 3’ ends of the most tRFs we analyzed (Fig. 4).

The results for the most abundant Ago2-loaded Gly-derived tRFs detected in our studies strongly supported the seed location on the 3’ end of tRFs (Fig. 4a-b, d-e). We observed that the tRF GlyGCC 7mer located at position 12 (to 18) has the highest frequency of reverse complement occurrences in the conserved regions of *Drosophila* genomes (regions associated with >14,000 genes in total), making it a candidate seed sequence (Fig. 4a, d). A very similar tRF, GlyTCC (attcccggccgaCgcacca), contained a one nucleotide difference to GlyGCC (attcccggccgaTgcacca) and had a candidate seed located at position 11 (to 17), shifted one nucleotide towards the 5’ end (Fig. 4b, e).

We found no overlap between the lists of *D. melanogaster* transcripts with matches to the seeds of GlyGCC compared to GlyTCC. Thus, although a single nucleotide difference in/near the seed region may influence tRF targeting and hybridization, it is remarkable that a very different set of conserved sequence matches/potential targets still corresponds to the same 3’ location of the seed sequence. Though many tRFs showed a peak similar to those in Fig. 4a-b, we noted that a few tRFs showed such peaks in the 5’ region, suggesting a 5’ seed targeting. For example, in the tRF mt:SerGCT the 7mer window matches peaked at the 5’ end of the sequence (Fig. 4c), as opposed to a 3’ end maximum found in the Gly-related tRFs. Thus we also observed potential seeds on both 5’ and 3’ ends of tRFs, in parallel to what was detected experimentally.

The enrichment in the counts of matches for potential seed sequences is very prominent in the conserved genomic regions (Fig. 4). The frequency of their 3’UTR matches far exceeds the expected frequency (between five and several hundred fold). At the same time, the seed matches are not among the most frequent heptamers in the *D. melanogaster* genome (e.g., the genomic frequency of the potential seed match in mt:SerGCT is less than half of the top heptamer). All these facts point to a possible functional role for the seed sequences, similar to those of miRNA seeds.

Intron sequences also produced much higher numbers of matches than expected (and also higher than reshuffled tRF 7mers), when conserved *Drosophila* introns were analyzed (Fig. 4d-f). In GlyTCC, the intronic matches even slightly exceeded the 3’UTR matches, although the major seed peaks in both cases were on the same 3’ end of the tRF (Fig. 4h). This may indicate targeting of common elements contained inside introns or a possible involvement of tRFs in the nuclear processes, as discussed below.

### Potential targeted regions in mRNA

**Fig. 5:**
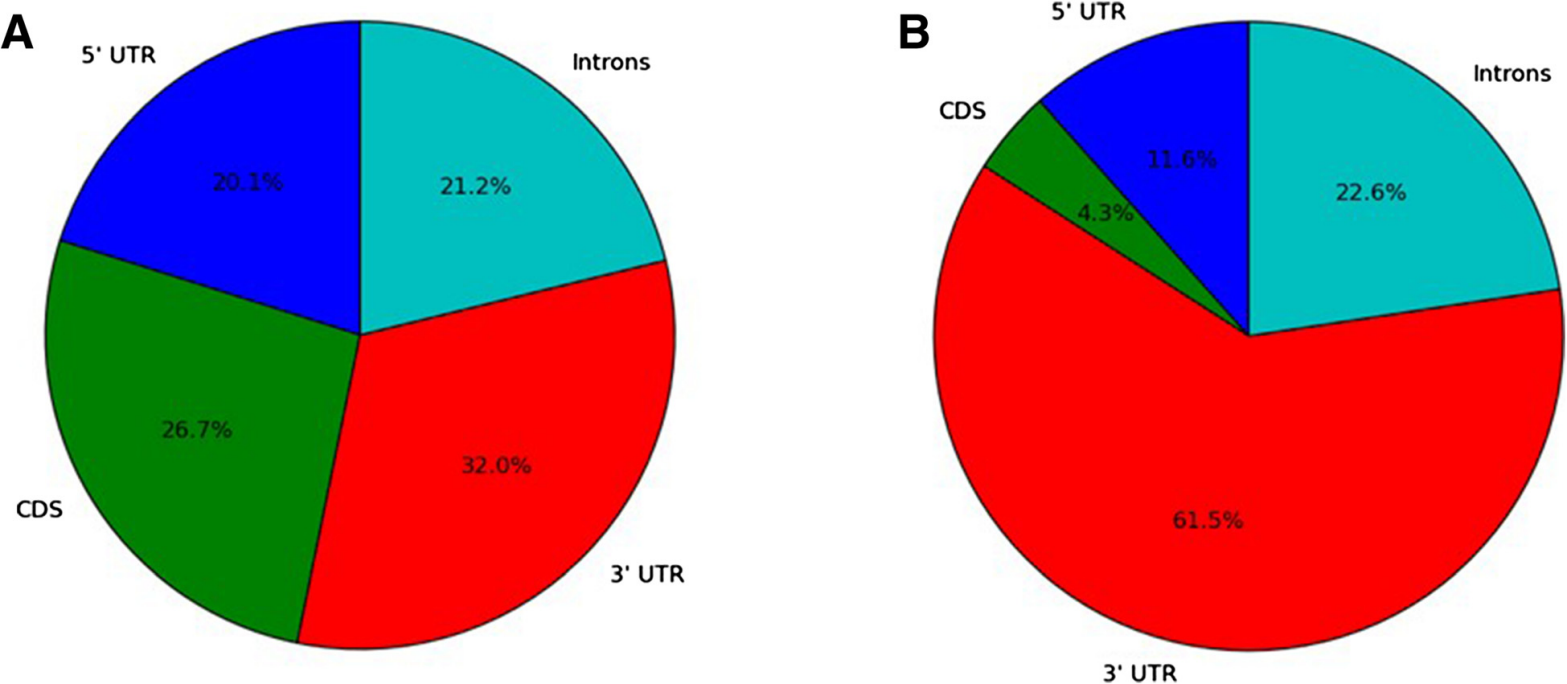
Share of Seed Alignments by Region. The relative share of seed matches to the most abundant 7mer (as shown in Fig. 4) of (**a**) GlyGCC and (**b**) mt:SerAGC

To find potential targeted regions, we compared the 5’ UTR, 3’ UTR, intron and exon parts of genes in the *D. melanogaster* genome. Per unit of length the 3’ UTR regions matched the potential tRF seeds most frequently (Fig. 5) suggesting a prevalence of a 3’ UTR targeting mode. This further supports the idea that tRFs may behave similar to miRNAs.

We observed significant enrichment of the 3’UTR for mt:SerGCT seed matches in the *D. melanogaster* genome (p < 0.001), both among random heptamers and among reshuffled nucleotides comprising the seed. The Gly tRF seed matches, with less extreme AT-richness, did not show such enrichment. However, we note that shuffling of the seed sequence is not an ideal random model and statistical testing of the tRF seed regions is complicated by the fact that a tRNA sequence is under multiple selective constraints for its structure and function related to translation (and furthermore different from the constraints of a miRNA).

We also scanned for nearly perfect complementary matching between full-length tRFs and 3’ UTRs, which would inform us if some of these tRFs acted like plant miRNA. This analysis, however, yielded no significant results, suggesting that the tRF binding mode may be more consistent with animal miRNAs.

**Fig. 6:**
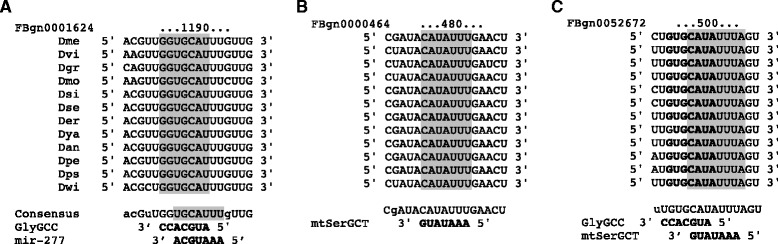
Examples of Seed Region Matches in Conserved 3’UTRs. Grey highlights and bold text indicate seed complementarity to conserved (12 *Drosophila* genomes) 3’ UTR regions. Targeted genes with overlapping coordinates in the genome are shown on top. **a** Both GlyGCC and mir-277 having a 7mer-m8 match, **b** mt:SerGCT 7mer-m8 match and **c** GlyGCC having a 7mer-1a match and mt:SerGCT having a 8mer-1a match, with additional A for the 1a matches are also highlighted (mt:SerGCT) or bolded (GlyGCC)

Assuming the latter (animal-like) binding mode, we observed a variety of seed sequence matches in the conserved fly genome regions. As with miRNAs, there were 7mer-m8, 7mer-1a and 8mer-1a match types. These types have been studied and confirmed previously for miRNAs [16] and are as follows. 7mer-m8 is a match of 7 nts (Fig. 6a and b). 7mer-1a and 8mer-1a refer to matches of first 6 (Fig. 6c, GlyGCC) or 7 (Fig. 6c, mt:SerGCT) nucleotides of the seed, respectively, followed by an extra A (added to the elongated match region in the Fig. 6c). All three illustrated targets (Fig. 6a-c) possess 3’UTR regions highly conserved amongst all 12 *Drosophila* genomes analyzed.

Notably, some seeds showed overlap with the seed of either another tRF or a miRNA (Fig. 6a and c). For example, both GlyGCC and mir-277 seeds overlap by 5 nts and this sometimes led to their complementarity against the same target (Fig. 6a). Such overlaps could theoretically lead to competition of tRFs and miRNAs for the same targets, potentially adding another layer of complexity to the regulatory processes.

As demonstrated by our results, there is clear evidence that tRFs interact and are loaded onto Argonaute proteins and may target the 3’ UTR regions of mRNAs, suggesting a potential post-transcriptional regulatory mechanism similar to that of miRNAs. The fact that the candidate seeds aligned predominantly to the 3’ UTRs indicates that one of the mechanisms for suppression may be translational inhibition. Alternatively, some tRFs may employ mRNA cleavage for regulation, since we observed CDS regions that also aligned to our candidate seeds [16–18].

### Gene ontology analysis of potential targets

Given the difference in seed localization, we predicted targets for the divergent cases of the Gly and mt:SerGCT tRFs. Following the link between the Ago-loading change of miRNA and brain degeneration with age [21], we assessed whether targets of these tRF were also associated with a particular biological process. Using the identified seed sequences, we sought targets for the tRFs in the *D. melanogaster* genome based on perfect matches to 3’UTRs. We then conducted a gene ontology (GO) enrichment analysis using the AmiGO 2 software [32] to understand the nature of the predicted targets.

Stringent criteria for enrichment revealed several interesting trends. Notably, neuronal and developmental processes were the most dominant among the significantly enriched terms (*p*-value < 0.001) belonging to the GO category “biological process”. In particular, for GlyGCC we observed 52 % of enriched GO terms related to development and 15 % related to neuronal function, while for mt:SerGCT these numbers were 39 % and 12 %, respectively (Additional file 1). In the GO analysis, the most populated process terms (if one counts potential targets, described by these terms) are often generic ones, like “biological process” or “biological regulation”. For both of these tRFs, the most populated GO terms after the generic ones were GO:0032502 (developmental process) or GO:0048856 (anatomical structure development). Pertinent to the tRF involvement in the neuronal regulation, synapse- or axon-related GO terms accounted for 20 % (in mt:SerGCT) to about half (in GlyGCC) of the significantly enriched terms (*p*-value < 0.001) in the category “cellular localization” (Additional file 2). The targets, exemplified in Fig. 6, belong to these GO categories, e.g., Dlar, a targeted gene of mt:SerGCT (Fig. 6b) is a conserved member of the tyrosine phosphatase family with a fundamental role in axon targeting/development and organization of actin filaments [33, 34]; see Discussion). For the category “molecular function”, terms related to DNA and RNA binding (with variations including regulatory region or nucleotide binding) were frequently enriched for mt:SerGCT and GlyGCC (Additional file 3).

Alternative polyadenylation and longer 3’UTRs have been observed in the transcripts in fly brains [35] and we checked if that affected our results. For all targets found above (using longest annotated 3’UTRs) we selected the shortest annotated 3’UTRs (if those were available) and again searched for matches to the seeds. As expected, there was a reduction in the numbers of both seed matches and corresponding targets. However, the reduction for the brain-associated genes (54.8 % of matches and 60.1 % of targets remaining) was very similar to the rest of tRF targets (53.4 % of matches and 61.3 % targets remaining) and this factor could not explain the GO term enrichment described above. As for the 3’UTR length itself, the coefficients of variation in both target sets were very high (both > 1) thus the length difference was not significant between these subsets of genes.

## Discussion

In this report we characterized tRFs found in Ago1 and Ago2 IP libraries from *Drosophila* to reveal expression and loading patterns in the context of age. We also identified potential targets and a likely mode for targeting.

We identified tRFs in both Ago1 and Ago2 co-immunoprecipitated libraries, indicating miRNA-like functionality of loading of these tRFs into RISC complexes. Alignment to the mature tRNA sequence revealed a high read-depth on one side of the tRNA molecule and size distributions of 16–30 base pairs in length, which suggests a similar structural motif as miRNAs. Although the library was size-selected for these distributions, we observed very precise boundaries of tRFs (similar to those in miRNA [21, 22]), strongly suggestive of a biological process rather than random degradation responsible for their generation. However, given the isoform diversity, limited degradation effects on the tRF ends cannot be ruled out, and their scale is comparable to “nibbling” in miRNA [21, 22].

By examining age-associated patterns of tRF expression, we saw distinct isoforms changes in age-dependent manner in *Drosophila*. For example, for GluCTC we observed the same isoforms present in both Ago1 and Ago2 libraries, but an increase in individual isoforms in Ago2, and a decrease in Ago1, especially for most abundant or major isoform. Additionally, the major isoform of AspGTC in Ago2 was not present at all in Ago1 (see Fig. 2). These types of change are correlated with a shift in loading of these fragments into Ago2 vs Ago1 with age. Thus, the partitioning of multiple tRFs between Ago1 and Ago2 may be a coordinated process modulated with age in *Drosophila* (see Fig. 3).

One possible explanation proposed for the observations of differential miRNA loading with age (which can be extended to tRFs) is that the cells are adjusting their regulatory processes for upcoming age-associated stresses [21]. Since Ago2-mediated translational silencing causes retention of the polyA tail [36], Ago2-association might make it possible to respond to age-associated internal or external stimuli more rapidly and effectively by allowing for re-activation of target mRNAs. Ago2 mutants have been shown to develop neurodegenerative phenotypes in the study of miRNA involvement in the aging process [21]. This may serve as further support of our predictions of the tRF regulatory function since in these mutants the disrupted stabilizing modification, lack of tRF RISC loading, and subsequent deregulation of the neuronal targets could further contribute to such phenotypes. One possible target of GlyGCC and mt:SerGCT (see Fig. 6c), the gene Atg8a, is intimately linked to aging pathways, e.g., the insulin/IGF-signaling pathway that mediates the lifespan in *Drosophila* through Smad binding [37].

Other modes of tRF-driven regulation have been proposed, from to inhibiting translation initiation factors to direct interaction with ribosome, etc. [2, 5, 7, 9, 12–14]. Given the base pairing in the tRNA stems, one cannot exclude potential interaction with full-length host tRNAs or their fragments. While this paper was under review, a possible role of tRFs as tumor suppressors binding to oncogenic RNA-binding protein YBX1, displacing pro-oncogenic transcripts has been described [38]. However, the patterns of conservations we observed indicate a clear possibility of miRNA-like targeting.

Although the exact mechanism is still being unraveled, our results suggest a short seed region in tRFs that is key for recognizing potential mRNA targets. While for animal miRNAs the 5’ seed location is most common, 3’-compensatory sites [29] and central pairing sites [30] have been reported. In our examples, the Gly-associated tRF in *Drosophila* has a putative 3’ seed region, while the mt:SerGCT tRF has a 5’ seed. Thus, in parallel to experimental data showing two possible seed locations [10, 15, 20], our results demonstrate that regions of conservation can be present at either the 5’ or the 3’ end in different tRFs. We also provide evidence that the 3’ UTR may be where targeting occurs, allowing us to speculate that the mode of action may include translational repression or mRNA cleavage.

Alternatively, some tRFs may employ mRNA cleavage for regulation, since we observed CDS regions that also aligned to our candidate seeds [16–18]. Enrichment of seed matches in the conserved intron regions may also indicate a role of tRFs in alternative splicing and transcriptional regulation, given the evidence of Ago2 involvement in these process in the nucleus [39]. The enrichment of targets involved in development may be of particular interest in this regard as Ago2 transcriptional target genes are also bound by Polycomb group transcriptional repressor proteins and change during development [39].

*Drosophila* Ago1 and Ago2 employ different mechanisms to silence target mRNAs and in particular Ago2 mutants show neurodegeneration and a shortened lifespan [21]. The fact that most tRFs are loaded and/or show a dramatic change in loading with age in Ago2 suggests that these small RNAs may also be involved in such pathways. In this regard, it is notable that despite the difference in seed localization (and no common targets), putative targets of tRFs from both mt:SerGCT and GlyCTC are significantly enriched in developmental and neuronal functions (Additional files 1: Table S1, 2: Table S2 and 3: Table S3). Further, we found that these target lists overlap (with up to 29 targets) with the well-studied miRNAs mir-34, mir-277, mir-190, and mir-10. All of these miRNAs impact brain function, affecting neurodegeneration, bi-polar disorder, and schizophrenia [22, 40, 41], in agreement with our predictions of tRF influence on the brain and age-related events. An overlap of the tRF seed with that of mir-277 is of importance, as it may relate one of the most abundant tRFs (GlyGCC) to brain deterioration, since mir-277 has been reported to modulate neurodegeneration [42].

Amongst the common targets of GlyGCC and mir-277, we observed Dlg (FBgn0001624), coding for the *Drosophila* discs large tumor suppressor protein (see Fig. 6a). The mechanism of regulation of this gene would be of interest since it has been previously associated with neuron development [43, 44] and it also shows homology with a human tumor suppressor protein [45]. Another common target, Toll-7 (FBgn0034476), may also be of significance, since it acts as a neurotrophin receptor and neurotrophism is only starting to be elucidated in insects [46].

Some of the tRF targets in the significantly enriched GO categories are closely related to the RNA regulatory pathways, e.g., Fmr1 (FBgn0028734, a homolog of the fragile X mental retardation 1 gene in human). This is an RNA-binding protein that interacts with the RISC complex itself and is necessary for proper development [47–49]. Of note, this gene is located in the *Drosophila* genome in the immediate vicinity (a few hundred basepairs) of mir-34 and mir-277, hinting at a potentially deeper regulatory connection.

## Conclusions

This is the first time such a detailed analysis has been performed on tRFs. We developed a robust pipeline to identify candidate “seed” regions that clearly showed a stronger binding pattern based on specific positions, restricting it to the 5’ or 3’ end and a binding preference for 3’ UTRs. The results reveal tRFs features that in many respects resemble structural and functional properties of miRNAs and strongly suggest that these small RNAs are not simply tRNA degradation products, but are specific, biologically-generated species. The targets predicted with candidate seeds showed enrichment in processes related to neuronal function and development, hinting at the biological significance of these tRF molecules. Thus, the trends observed with tRFs likely represent bona fide targeted processing of tRNAs, and the tRF association with different RISC complexes in the context of age may reflects an important regulatory function.

## Methods

### Mapping and quantifying tRFs

We used *Drosophila* Ago-IP libraries GSM1278635, GSM1278636, GSM1278637 and GSM1278638 available from the Gene Expression Omnibus (GEO) database, with experimental details described earlier [21]. Adaptor sequences were removed from the 3’ end of the reads in the Illumina fastQ files using the fastx-toolkit (http://hannonlab.cshl.edu/fastx_toolkit/). The adapter sequences are as follows:

- 5’ adapter = 5’- GUUCAGAGUUCUACAGUCCGACGAUC- 3’
- 3’ adapter = 5’- TGGAATTCTCGGGTGCCAAGG- 3’

Reads were then collapsed and annotated with the number of times each was sequenced, so only unique reads were analyzed. The reads were then mapped using Bowtie to the *D. melagonaster* (dm5) genome and tRNAs obtained from FlyBase. Bowtie parameters were restricted to only output perfectly aligned matches to the tRNA sequence. The reads were aligned and mapped to the entire tRNA sequence with the CCA addition. After mapping reads to their respective tRNAs, each library was independently normalized by the total number of reads mapped to the *D. melanogaster* genome (v. R6.03).

### Differential/preferential loading with age

We identified differential loading of tRFs with age in Ago1 and Ago2 using a ratio metric. We first identified the most abundant isoform in our 30 day libraries and used the read count numbers of that specific isoform for our ratio calculations. We calculated the ratio of 30 days to 3 days for Ago1 and Ago2 of highly abundant (1000 or more reads) tRFs. We then plotted the ratios to see loading changes that may occur with age.

To observe what was preferentially loaded (Ago1 vs Ago2) with age, we obtained a different ratio. The ratio of this measure was the ratio of reads of a particular tRF of Ago2 to Ago1 at 3 days and at 30 days.

### Analysis of seeds, targeting and GO terms

In order to identify a potential seed sequence in our dataset, we generated *k-*mer subsequences of the tRF by applying a sliding window by shifting one nt towards the 3’ end after each subsequent *k-*mer generation. We then found exact matches for each of these subsequences to the conserved 5’ UTR, 3’ UTR, exon and intron regions of 12 *Drosophila* genomes provided by UCSC [50] and to those regions in the *D. melanogaster* genome. We then compared for each *k-*mer in a tRF the observed number of its matches in the conserved 3’ UTR regions with the expected number (based on the frequency of matches across the *D. melagonaster* genome) and with the average number of matches of all possible *k-*mers with the same nucleotide composition in conserved 3’UTRs to identify candidate seeds. Genes with exact matches of 7mer candidate seeds to the longest annotated 3’UTR were considered potential targets. While our approach is similar to TargetScan [16–18], we did not use its “context score” as it was unlikely to be applicable for our cases of both 3’ and 5’ seeds. To find the preferentially targeted regions we normalized the total match counts by the total length of each respective set of regions. AmiGO [32] was used to find enriched GO-terms in our target list for each tRF.

## Reviewer’s comment

### Reviewer’s report 1: Dr. Eugene Koonin, NCBI, United States of America

This is a very interesting, provocative piece of analysis that suggests distinct, age-dependent regulatory roles for tRNA fragments. The age-dependent association of tRFs with Ago1 and Ago2 is highly suggestive of functional importance. The analysis of seed sequences is interesting but I think it is desirable to present statistical argument for the validity of the identified seeds. I have not understood the argument on the protective role of O-methylation. Is this based on a single abundant tRF?

On the whole, I think that in places, the article is assertive beyond what the observations justify (eg the last sentence in the Background section). To me, the regulatory function of tRFs remains a hypothesis, even if one that is compatible with an impressive body of observation. More on the semantic side, the authors repeatedly write about confirming or supporting hypotheses which does not seem to be good practice. “Compatible with the predictions” is better phrasing.

Author’s response: Following these constructive suggestions, we have added more data for the seed sequences, toned down some of the assertions and rephrased several sentences. We have removed the text on the possible role of protective 2-O-methylation of tRFs with age given the limited experimental data on oxidized RNA available.

### Reviewer’s report 2: Dr. Neil Smalheiser, University of Illinois at Chicago, United States of America

This article provides an extensive analysis of tRNA fragments in Drosophila that provides strong, but indirect, evidence that they play miRNA-like roles. In general, the data are presented in an anecdotal rather than a rigorously statistical fashion, which may fail to convince many readers.

Specific comments:

- p. 5. The study is a re-analysis of datasets that are only mentioned in passing here. It would be helpful to give more information in Methods about the datasets, how they were produced, what kind of controls or validation ensure their reliability, etc.
- p. 6. It is claimed that tRNA fragment cleavages are non-random and precise, but in part, that is because one end is the 5’ or 3’ terminus! That is not the same as precise cleavage at both ends. In fact, one end generally shows a lot of variable processing - which may be similar to the variable processing of miRNAs, yet the ends are not so precise that one can rule out degradation (cf. miRNAs which are subject to nibbling).
- p.6. It is nice that the data used for analysis have been placed on the authors’ website, but this is not satisfactory for several reasons. First, the data are not well described or cited on the website. Second, there is no version control. There is no indication whose data these are, or what the terms of use are. Third, and most important, this is NOT a repository - there is nothing to prevent the link from being removed or modified at any time. They should deposit the data in a permanent location, either a university repository, or one of the growing open data repositories.
- p.7. Amongst tRFs with counts > 100?. How was this value chosen?
- p. 9–10. 7-mer window method and findings are not precisely described.
- p.11. Significant enrichment of the 3’-UTR for mt:SerGCT seed matches in the genome, both among random heptamers and among reshuffled nucleotides. This is the first statement that employs statistics with baseline datasets. I agree that shuffling seeds alone is not compelling, but this is the kind of data you want more of, and is missing from much of the paper.

It appears you only compare 5’-UTR, CDS and 3’-UTR regions of mRNAs in your analyses - what about introns? Long ncRNAs? Non-genic sequences? Genomic repeats? Are they negative or do they contain putative targets too? Won’t the host tRNA genes have some complementarity and be putative targets?

miRNAs tend to exhibit multiple ‘hits’ with the same and different miRNAs, near each other, on a given mRNA target. Do you observe this same phenomenon with tRNA fragments?

- p.15. Discussion. The data regarding 3’-protected tRNA fragments is equivocal and might be omitted.
- p.16. Brain mRNAs tend to have very long 3’-UTRs. Is that a possible confound in the interpretation of your finding that putative tRNA fragments tend to target them?
- p.25. Fig. 1. Would be nice to see the actual folding of the tRNA, especially to visualize the exact length and pairing of the 5’ and 3’ stems.

There is A and B in the figure but not mentioned in the legend.

Fig. 4. The number of conserved sequence matches are shown, but this is not very helpful to the reader. Certainly different fragments will match different numbers of sequences in different places. But what number of matches are expected by chance? How over-represented are these matches relative to what might be produced by some shuffled sequences, or some non-physiological set of sequences with similar length and dinucleotide composition, etc.?

Fig. 6. Since tRNAs are highly conserved in a variety of functions, the fact that seed sequences are conserved is not compelling, unless a) they reside in regions of the tRNA that otherwise lack known functions, or b) ONLY the seed regions are conserved and not the flanking regions. This issue needs better discussion and analysis.

Figures 1 through 5 are not well done in terms of conveying the main points directly to the reader without needing to read the legends in detail. In Fig. 3, a critical point appears to be whether a given bar is less than one or greater than one, but ‘one’ is not shown or legible in the graph. In Fig. 5, it is not clear whether the pie charts have taken into account the fact that 5’-UTRs, CDS and 3’-UTRs tend to be of different lengths. Finally, the Additional files lack any description or legends within the manuscript.

Author’s response: We thank the reviewer for providing very specific comments, which we have tried to address in the revised version. We have corrected small omissions and typos in the text, figure legends and additional files. We have modified nearly all figures adding color and additional details and figure panels to clarify our points. We have added to the description of the seed finding. An example of multiple tRF ‘hits’ of the same gene (Atg8a) is given and commonality of tRF targets with miRNAs is discussed.

We have provided further details of the sequencing data. These datasets are available from the NCBI, unlike our results processed for display on our lab website – to our knowledge there is no public repository that can present them in this form. The horizontal display does present the secondary structure/folding details (with shaded stems shown right next to the fragments), albeit not in the traditional form, but we have added to Fig. 1 to point to the locations of the specific tRNA loops in that display for clarity.

We agree with the reviewer that some degradation effects on the tRF ends cannot be ruled out. Further, even tRNA ends do show variability (and Fig. 1 also illustrates that) to the extent is comparable to “nibbling” in miRNA – we have added this to the Discussion.

We have removed the text on the possible role of protective 2-O-methylation of tRFs with age given the limited experimental data on oxidized RNA available. Indeed, a single isoform of GluCTC is not sufficient to make a statement beyond an anecdotal observation.

We have extended our comparisons to the 5’ UTRs, CDS, intronic and 3’UTR regions. We have added text to the corresponding section and changed the Fig. 4 to compare the observed counts with those expected by chance or for reshuffled sequences. Indeed, in our comparisons we have found significant enrichment for introns and we have further added to our discussion of possible targets or functional role of tRFs.

We have modified the legend of Fig. 5 to clarify that a share of matches per unit length is shown. We have modified the legend of Fig. 6 to clarify the illustration of the conserved 3’UTR rather than a conserved tRF sequence.

### Reviewer’s report 3: Dr. Alexander Kel, geneXplain GmbH, Germany

The manuscript of Naqvi and coauthors: “Age-driven modulation of tRNA-derived fragments in Drosophila and their potential targets” is very important for further understanding of diversity of molecular mechanisms of gene regulation. Regulatory role of some tRNA fragments (tRFs) was shown before, providing various evidences for the mechanism of action and pathways involved in the tRNA processing, but for me it was always considered as some sort of a curious thing which was taken by evolution and used for regulating some minor processes in the cell.

Whereas, the current paper has demonstrated that these fragments seemingly play an important role in regulation of many processed and that there is a highly specialized mechanism of preprocessing of these fragments for their further use in gene regulation. It also looks very logical that the fragments of such abundant RNA molecules in the cell as tRNAs are picked up by the evolution and applied for the regulation of various processed especially since existence of such powerful mechanism as RISC system for the regulatory usage of small RNA molecules. Moreover, due the relation of tRF molecules to the tRNAs, I will not be surprised if one day researchers will find connection between regulation of translation efficiency due to the differential regulation of abundance of different tRNA species and regulation of the same genes (or genes of the same pathways) by the tRFs derived from the respective tRNAs.

For me it was also very interesting and somewhat surprising to see that authors observed that seeds (of complementarity to the mRNA targets) could be fond both on 5’ as well as 3’ ends of the tRF molecules. It tells me about either very ancient mechanism of processing of these molecules or about possible artefacts in these observations (for instance due to the low sequencing counts). I think, more detailed analysis of this fact is necessary to validate this finding. Also, considering the potentially ancient nature of these regulatory mechanisms it is really interesting and surprising to see that the most prominent GO terms enriched by the mRNA targets of tRFs were belong to neuronal function and development, which are quite late events in the evolution. This only confirms that the nature of gene regulation is still full of surprises and that bioinformatics approaches often play an important role in novel discoveries of basic principles of organization of biological systems.

Author’s response: We thank the reviewer for these comments, placing our observations into a broader regulatory context. The possibly ancient nature of the mechanism may seem to be at odds with the neuronal targets, whose function is a relatively recent evolutionary invention, but this may also point to a flexible or opportunistic character of the regulatory mechanisms utilizing small RNA fragments.

## Additional files

Additional file 1:
**Significantly enriched GO terms in the analysis of potential targets of GlyGCC.** AmiGO [32] output (showing enrichment of specific GO terms) is provided for the three GO catgories in three respective worksheets. (XLSX 8 kb) Additional file 2:
**Significantly enriched GO terms in the analysis of potential targets of GlyTCC.** AmiGO [32] output (showing enrichment of specific GO terms) is provided for the three GO catgories in three respective worksheets. (XLSX 12 kb) Additional file 3:
**Significantly enriched GO terms in the analysis of potential targets of mt:SerGCT.** AmiGO [32] output (showing enrichment of specific GO terms) is provided for the three GO catgories in three respective worksheets. (XLSX 16 kb)

## Abbreviations

tRF: Transfer RNA fragment
tsRNA: tRNA-derived small RNA
ncRNA: Non-coding RNA
UTR: Untranslated region
RISC: RNA-induced silencing complex
GO: Gene ontology
